# Comparison of Phenotype Nutritional Profiles and Phosphate Metabolism Genes in Four Serovars of *Salmonella enterica* from Water Sources

**DOI:** 10.3390/microorganisms11082109

**Published:** 2023-08-18

**Authors:** Lisa Gorski, Ashley Aviles Noriega

**Affiliations:** Produce Safety and Microbiology Research Unit, Agricultural Research Service, United States Department of Agriculture, Albany, CA 94710, USA

**Keywords:** *Salmonella*, serovars, growth, nutrient utilization, phosphate, phenotype microarray, PhoR

## Abstract

The surveillance of foods for *Salmonella* is hindered by bias in common enrichment media where serovars implicated in human illness are outgrown by less virulent serovars. We examined four *Salmonella* serovars, two common in human illness (Enteritidis and Typhimurium) and two that often dominate enrichments (Give and Kentucky), for factors that might influence culture bias. The four serovars had similar growth kinetics in Tryptic Soy Broth and Buffered Peptone Water. Phenotype microarray analysis with 950 chemical substrates to assess nutrient utilization and stress resistance revealed phenotype differences between serovars. Strains of *S.* Enteritidis had better utilization of plant-derived sugars such as xylose, mannitol, rhamnose, and fructose, while *S.* Typhimurium strains were able to metabolize tagatose. Strains of *S.* Kentucky used more compounds as phosphorus sources and grew better with inorganic phosphate as the sole phosphorus source. The sequences of nine genes involved in phosphate metabolism were compared, and there were differences between serovars in the catalytic ATP-binding domain of the histidine kinase *phoR*. Analysis of the predicted PhoR amino acid sequences from additional *Salmonella* genomes indicated a conservation of sequences each within the Typhimurium, Give, and Enteritidis serovars. However, three different PhoR versions were observed in *S.* Kentucky.

## 1. Introduction

The most common agent of foodborne illness is non-typhoidal *Salmonella enterica* [1,2]. Recency-weighted statistical modeling attributes most salmonellosis as resulting from contaminated seeded vegetables, eggs, chicken, produce, and beef [3]. There are over 2650 different serovars of *Salmonella*, but most human illness is caused by approximately 100 serovars, with the U.S. Centers for Disease Control and Prevention recognizing the top 20 serovars implicated in human illness in annual reports. The serovars Typhimurium and Enteritidis are among the top 5 causes of salmonellosis in the U.S. and the top 2 in the European Union and China [4,5,6]. Contamination of foods and water with multiple serovars is not uncommon. Using CRISPR-SeroSeq, Cox et al. reported an average of five different *Salmonella* serovars present on broiler carcasses [7]. A recent outbreak of salmonellosis in France associated with dried pork sausages was traced back to two different serovars, Bovismorbificans and monophasic Typhimurium [8]. Similarly, several surveys of surface water report the isolation of multiple serovars of *Salmonella* from the same sample [9,10].

Laboratory methods for *Salmonella* surveillance and traceback investigations of *Salmonella* outbreaks involve suspension of the food sample in a primary enrichment or pre-enrichment medium and incubation for 18–24 h at 35 °C–37 °C, followed by secondary enrichments and plating on various selective and differential media. Standard methods used in various surveillance laboratories prescribe several different complex, liquid media for pre-enrichments and primary enrichments [11,12,13]. Standard pre-enrichment media are dilute, complex media that are based on the protein hydrolysates of casein or peptone that allow for the recovery of stressed and injured cells that might be present in contaminated foods [11,12,14]. Buffered Peptone Water (BPW) is recommended in the methods published by the USDA’s Food Safety and Inspection Service (USDA-FSIS) and the International Organization for Standardization (ISO) to pre-enrich *Salmonella* from chicken and other foods [11,12,13]. Tryptic Soy Broth (TSB) is a common peptide-based, laboratory growth medium that is routinely used for *Salmonella* growth and is also used in non-selective enrichment of *Salmonella* from eggs and spices according to the Food and Drug Administration (FDA) method [11].

Culture bias in laboratory media influences the serovars of *Salmonella* that arise from samples containing multiple subtypes [7,15,16]. The *Salmonella* serovar Kentucky is prevalent in poultry in the U.S. and is frequently isolated in *Salmonella* enrichments, where it outgrows strains of serovars such as Typhimurium and Enteritidis [7,17,18]. Similarly, *S.* Give is frequently isolated from surface water and from ruminants [9,19,20,21,22]. The bias can be problematic when conducting food surveillance or outbreak tracebacks because the serovars Kentucky and Give are rarely associated with human illness in the U.S. [4]. In the U.S., *S.* Kentucky and *S.* Give are more fit in commonly used *Salmonella* enrichment protocols and can outgrow and mask the presence of strains of *S.* Typhimurium and *S.* Enteritidis [7,15,22,23]. In Europe, the Middle East, and several African countries, an antibiotic-resistant *S.* Kentucky Sequence Type 198 has arisen to cause multiple cases of human illness. While ST198 has caused human illness in the U.S., the number of cases is markedly lower [24,25].

In BPW and TSB, peptides serve as the sources of carbon, nitrogen, sulfur, phosphorus, and energy for the microbiota. Differences between strains in the utilization of media components may affect the final recovery of strains in the mixture. These differences may be intensified when nutrients affect many roles in cellular metabolism. For example, proteomic analysis of the *S.* Typhimurium strain 14028s revealed over 300 proteins that are regulated by phosphorus-limiting conditions [26].

Phosphorus metabolism via the uptake of inorganic phosphate (Pi) has effects on magnesium and calcium homeostasis [27,28]. In *Salmonella,* phosphate homeostasis is controlled by protein synthesis by responding to reductions in ATP, destabilized ribosomes, or low cytoplasmic magnesium [29]. Phosphate levels are sensed and controlled through the actions of PhoB/PhoR, a two-component regulatory system in which PhoR is an integral membrane-bound sensor histidine kinase, which autophosphorylates when Pi levels are low, and transfers that phosphoryl group to PhoB. PhoB is a transcriptional regulator responsible for the moderation of gene expression of Pho genes. One of the operons affected by PhoB is the *pstSCAB* operon, which encodes a high-affinity ABC-type transporter responsible for the transport of Pi into the cell [30,31,32]. When intracellular Pi concentrations are high, the transporter is turned off through the interaction of the PhoU protein with the PstB component of the transporter. This PhoU–PstB interaction is mediated through the action of the sensor kinase PhoR [29,31]. Also, during periods of low Pi, the Pho regulon plays a role in maintaining the stability of the stationary phase and stress response sigma factor RpoS through the actions of the small protein IraP. IraP assures the continued action of RpoS by binding to and inhibiting the RssB adaptor protein so that RssB is unable to bind to RpoS and target it for degradation [30,32,33].

In addition to phosphate metabolism, low nutrient conditions induce ppGpp, which slows protein synthesis and may induce a stress response [34]. Differences between strains in the utilization or efficiency of utilization of basic nutrient compounds result in fitness variances based on the competition for nutrients and lead to differences in recovery of subtypes [35,36]. It is possible that minor differences in the sequence of proteins used for nutrient utilization may affect efficiency of action, which in turn might influence recovery in mixed cultures.

A systematic study of the nutritional capacity of *Salmonella* serovars is needed to assess potential nutrient utilization differences between serovars. This information would be helpful in the design of culture media to bolster poor performers such as Typhimurium and Enteritidis and/or lower the competitiveness of good performers such as Kentucky and Give during routine surveillance of food and water. Similarly, the genetic differences resulting in such nutritional differences also need investigation.

## 2. Materials and Methods

### 2.1. Strains and Growth Conditions

The strains used in this study are listed in Table 1. Most of the strains were isolated in our laboratory as part of a large surface-water survey [10]. Strains were chosen based on the serovar, and strains within serovars were of different Pulsed Field Gel Electrophoresis (PFGE) pulsotypes isolated at different times and from different locations. Serovar identity was previously determined using sero-agglutination [10]. Because *S.* Kentucky strains were not well represented in that survey, additional *S.* Kentucky strains from the strain collection in the Produce Safety and Microbiology research unit were added. In addition to the strains used for growth and nutrition experiments in Table 1, genome information for additional analysis was downloaded from Enterobase [37,38].

For general use, strains were routinely grown in Trypticase Soy Broth (TSB, Difco, Becton Dickinson, Franklin Lakes, NJ, USA) and Trypticase Soy Agar via incubation overnight at 37 °C. Liquid cultures were incubated in a shaking incubator (Infors, Annapolis Junction, MD, USA) set to 150 rpm. The Phosphate Buffered Saline (PBS) contained 150 mM of NaCl and 10 mM of sodium phosphate, pH 7.2.

### 2.2. Growth Kinetics

Growth kinetics of individual strains were measured in TSB and BPW (Difco). Single colonies were inoculated into 4 mL of TSB or BPW and incubated with shaking for 16 h–18 h at 37 °C. A portion (1.0–1.5 mL) of the resulting culture was centrifuged at 13,000× *g* in a microfuge for 3–5 min, and the cell pellets were resuspended in PBS to an A_600_ of 0.05–0.1. This cell suspension (35 µL) was used to inoculate 35 mL of fresh, room-temperature medium, resulting in a starting cell concentration of approximately 10^5^ CFU/mL. The TSB and BPW cultures were incubated with shaking at 37 °C. Turbidity was monitored by measuring A_600_ at regular intervals. Cell density was monitored by making serial dilutions of cultures in PBS and plating onto TSA. Plates were incubated for 18 h–24 h at 37 °C, and the colonies were counted to calculate the CFU/mL. These values were log-transformed, and the data were plotted as log CFU/mL.

Growth kinetics were modeled with a modified Gompertz equation using Prism 9.5 (GraphPad, San Diego, CA, USA) [39]. Growth curves were generated, and non-linear regression was performed using the Gompertz option in Prism. Best-fit values were derived from the curves in Prism according to Equation (1):

Y = Y_M_ × (Y_0_/Y_M_)^exp(−K × X) (1)

where Y_0_ is the starting population, Y_M_ is the maximum population level, K determines the lag time, and 1/K is the X value of the curve inflection point. Therefore, the 1/K value represents the duration of lag phase. Generation time was calculated during lag phase using the following equation:

g = t/n(2)

where g is generation time, t represents the time interval, and n is the number of generations within the time interval. The number of generations within a time interval calculated from the growth curves using the following equation:

N_t_ = N_o_ × 2^n^ (3)

where N_t_ is the cfu/mL at time t, N_o_ is the cfu/mL at time 0, and n is the number of generations during the time interval.

### 2.3. Phenotype Microarrays

Metabolic characteristics of selected strains were determined using phenotype microarrays (Omnilog, Biolog, Hayward, CA, USA). Bacteria were grown on Biolog Universal Growth Agar with 5% sheep blood, and bacteria were harvested and inoculated into Phenotype Microarray (PM) plates according to the manufacturer’s instructions [40]. PM plates 1–10 were used to assess the phenotype characteristics of strains RM15596, RM17366, RM18387, RM18411, RM18433, RM18465, RM19068, and RM20875 using pre-configured 96-well plates (Biolog). PM plates 1–10 measured growth resulting from 950 different compounds to test for metabolism of carbon sources (PM-1 and PM-2), nitrogen and peptide nitrogen sources (PM-3, PM-6, PM-7, and PM-8), phosphorus and sulfur sources (PM-4), nutritional supplements (PM-5), osmotic and ionic effects (PM-9), and pH effects (PM-10) [40]. Phenotypes for each strain were determined in three replicate experiments and analyzed using Data Analysis v. 1.7 software (Biolog). Data were expressed as Omnilog Units (OUs), which are a spectrophotometric-based measure of metabolism based on the intensity of a redox dye in each well of the PM plates. The range of OUs is 0–500. Each plate contained a negative control well to serve as a background value. Results for strain replicate experiments were compared to ensure reproducibility, and then strains and serovars were compared with each other using the Data Analysis v. 1.7 software. The background values were subtracted from the experimental OU values, and averages and standard deviations were calculated in the Data Analysis v. 1.7 software. The OU values of the wells for each compound were compared between serovars. For comparison purposes, the average OUs for each well were differentiated into three levels: <20 OUs, indicating little to no utilization; 20–100 OUs, indicating moderate levels of utilization; and >100 OUs, indicating high levels of utilization. Levels such as these have been used for phenotype microarray analysis by other investigators [41,42].

### 2.4. Genome Sequencing and CRISPR Sequence Comparisons

The genomes of RM15596, RM17366, RM18387, RM18411, RM18433, RM18465, RM19068, and RM20875 were sequenced using the Illumina Mi-Seq platform with 500-cycle v2 paired-end sequencing kits (Illumina, San Diego, CA, USA). DNA libraries were prepared and quantified using the KAPA Low-Throughput Library Preparation Kit and KAPA Library Quantification Kit (Roche, Kapa Biosystems, Wilmington, MA, USA) with modifications as described in [43] and validated on Agilent 2100 Bioanalyzer chips using the Agilent 12,000 kit (Agilent, Santa Clara, CA, USA). Sequences were trimmed and draft sequences were uploaded to Enterobase, where sequence quality was assessed, de novo assemblies were constructed, and the seven-gene Multi-Locus Sequence Type (MLST) was determined [37,38]. Serovars were confirmed with SeqSero2 [44]. Sequence coverage ranged from 67X to 300X. Genomes were annotated in RAST v2.0 (https://rast.nmpdr.org/rast.cgi, accessed on 2 May 2023) [45].

Genome sequence data were deposited with the NCBI under the BioProject ID PRJNA875079. The raw sequencing data were sent to the Sequence Read Archive and are identified by the BioSample accession numbers SAMN30597303–SAMN30597310. Strains were identified by their ID numbers: RM15596, RM17366, RM18387, RM18411, RM18433, RM18465, RM19068, and RM20875.

### 2.5. Gene and Protein Sequence Alignments

The DNA and predicted protein sequences of several genes involved in inorganic phosphate metabolism were compared. The identity and function of the genes of interest are shown in Table 2. The sequences of *phoB*, *phoR*, *phoU*, *phoP*, *phoQ*, *iraP*, *pstA*, *pstB*, *pstC*, *pstS*, *rpoS*, and *rssB* from the sequenced strains (indicated with an asterisk (*) in Table 1) were imported into Geneious Prime. DNA and predicted protein sequences were aligned using Geneious and/or MegAlign Pro v. 15.0 (DNAStar, Inc., Madison, WI, USA).

Thirty-eight additional *Salmonella* Enteritidis, Give, Kentucky, and Typhimurium genomes were used for added analysis of *phoR* sequences. Some of the additional genomes were from *Salmonella* strains in our collection that were previously sequenced [10]. Additional *Salmonella* genomes were downloaded from Enterobase [37,38]. The additional genomes used are indicated in Supplementary Table S1. Genome sequences were annotated in RAST, and the *phoR* gene sequences were determined. Alignments of predicted PhoR amino acid sequences were conducted in Geneious Prime.

## 3. Results

### 3.1. The Serovars Had Similar Growth Kinetics in TSB and BPW

**Figure 1:**
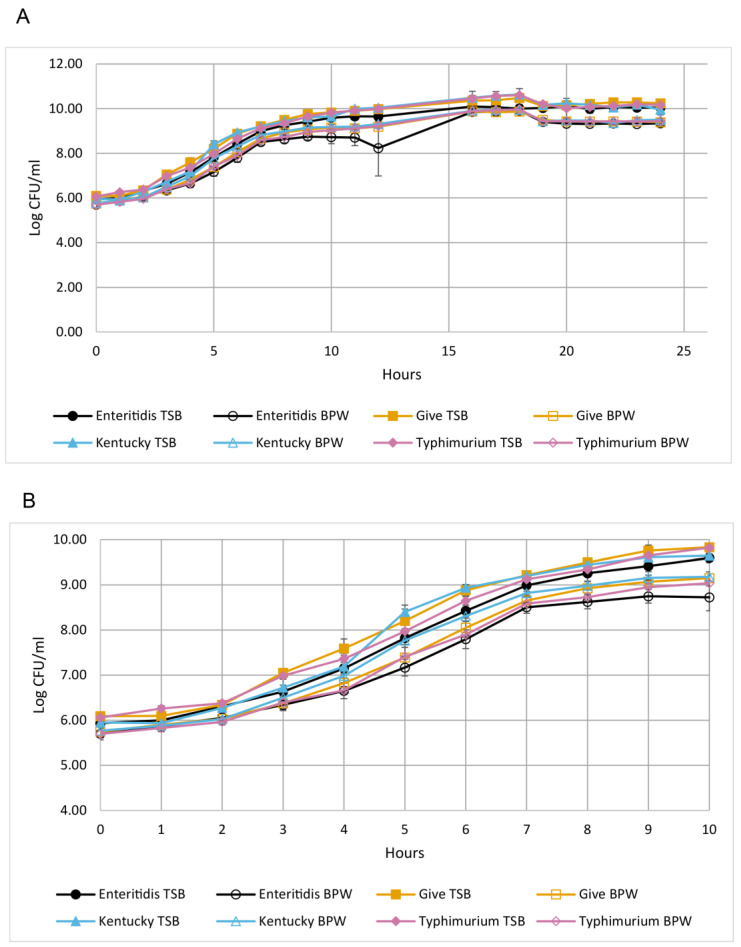
Growth curves of the Salmonella serovars in TSB and BPW. Growth data from the Salmonella strains were averaged and are displayed according to serovar. (**A**) Log CFU/mL over 24 h; (**B**) the same graph as in (**A**) but showing only the first 10 h to highlight the lag and log phases. Enteritidis is shown with black circles, Give is shown with orange squares, Kentucky is shown with blue triangles, and Typhimurium is shown with pink diamonds. TSB curves are solid lines, and BPW curves are dashed lines.

Growth in TSB and BPW was measured in the strains indicated in Table 1. Growth curves over 24 h are shown in Figure 1A. Figure 1B shows the first 10 h of the total 24 h growth to highlight the lag and log phases. The data for the individual strains were grouped according to serovar. The growth curves had similar shapes to each other, and the maximum CFU/mL numbers reached were similar for the serovars in pure culture in each medium. Non-linear regression analysis was performed on the growth data, and the model revealed that the serovars grew at similar rates in both media (Table 3). The lag phase in the Enteritidis strains was longer than in the other serovars (3.0 h vs. 2.3 h). Generation times were similar in TSB and BPW at approximately 0.5 h, with serovars Enteritidis and Typhimurium growing approximately 10% faster in pure cultures. The CFU/mL counts were approximately 1 log lower in BPW, consistent with BPW being a more dilute medium than TSB.

### 3.2. The Serovars Had Phenotype Differences in Nutrient Utilization

**Figure 2:**
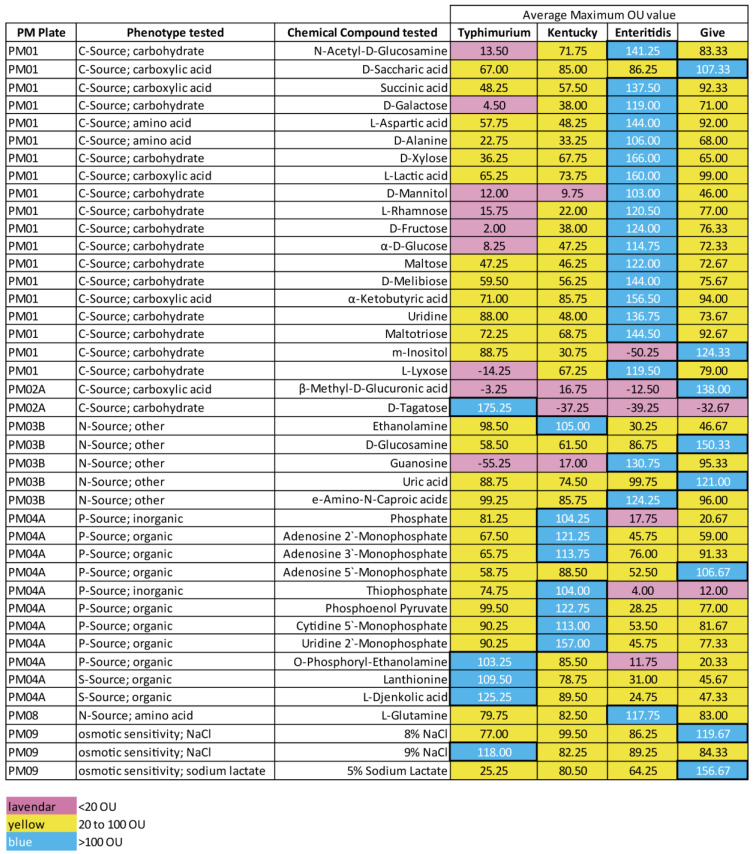
Heat map of 41 phenotype differences between the serovars. Strains were analyzed via phenotype microarray at least three times and background-subtracted, and the averages for strains were grouped into serovars. The numbers (OU range 0–500) are a measure of intensity of redox dye that reflects usage of the compound tested. The type of pre-configured microplates tested, the phenotype tested, and the chemical compound in the well are indicated. Cells are colored to indicate ranges of OU values (<20 OUs: lavender; 20–100 OUs: yellow; >100 OUs: blue).

A phenotype microarray analysis with pre-configured 96-well plates was used to assess the effect of and/or utilization of 950 compounds for carbon, nitrogen, phosphorus, and sulfur sources, as well as nutritional supplements and osmotic and pH tolerance. The phenotypes demonstrated in the wells of the plates was reflected by redox dye intensity as explained in the Section 2 and were measured in Omnilog Units (OUs), which range from 0–500. For analysis purposes, OUs were differentiated into three categories: little to no growth (<20 OUs), intermediate growth (20–100 OUs), and high growth (>100 OUs). The data were examined according to the individual strains and average of each serovar. Under these criteria, there were 157 wells among the 960 analyzed that showed differential OU categories between the serovars (Supplementary Table S2). These could be further differentiated by of the number of OUs between the different levels. There were 125 wells where there was >100 OUs between the highest utilizing serovar for a compound and one of the other serovars. A total of 84 of these wells measured nitrogen utilization of dipeptides, 70 of which had the highest levels of utilization (OUs ranging from 100–130) in serovar Enteritidis strains. The remaining 41 phenotype wells that showed differential utilization levels according to serovar are shown in Figure 2. The data for the individual strains are given in Supplementary Table S2. Of the 41 traits, 21 were carbon sources, 6 were nitrogen sources, 9 were phosphorus sources, 2 were sulfur sources, and 3 measured osmotic sensitivity. *S.* Enteritidis strains had the highest OU numbers among the carbon sources, showing utilization of several compounds that were not used by at least one of the other serovars. These included plant-derived sugars such as xylose, mannitol, rhamnose, fructose, and melibiose. *S.* Typhimurium strains showed little utilization of 8 of the carbon sources among this set of 21. However, *S.* Typhimurium were the only strains positive for utilization of D-tagatose, which is found in dairy products and can be derived from lactose. There was variability between the serovars regarding the non-peptide sources of nitrogen in the compounds shown in Figure 2. The average values of the *S.* Kentucky strains were the most diverse among the four serovars in the use of phosphorus sources, showing more or faster growth with inorganic phosphate, thiophosphate, phosphoenol pyruvate, and several nucleotide phosphates as the sole phosphorus sources. The *S.* Kentucky strain RM16068 had higher utilization of the phosphorus compounds than did the *S.* Kentucky strain RM17366, but both strains performed better than strains of the other serovars for these compounds (Supplementary Table S2). Due to the better performance of the *S.* Kentucky strains in the phosphate wells and the importance of the Pi utilization to several aspects of metabolism, we examined the phosphorus assimilation genes in the strains of different serovars.

### 3.3. PhoR Gene and Amino Acid Sequences Differed between Serovars

The predicted protein sequences of the *phoB, phoR, phoU, pstA, pstB, pstC, iraP, rpoS,* and *rrsB* genes among strains of the different serovars were compared, and the lengths of the nucleic acid and predicted protein sequences were conserved. For the sequenced strains in this study (those with an asterisk (*) in Table 1), the predicted amino acid sequences of PhoU, PstB, PstC, IraP, RpoS, and RrsB were identical (Supplementary Figure S1). Five of these six genes had SNP differences between the eight strains and four serovars (Supplementary Figure S2), but the changes resulted in the same predicted protein sequence. The DNA sequences for the *iraP* gene were identical in all the strains (Supplementary Figure S2). Among these six genes, the largest number of SNP differences were in *phoU*, which ranged from 0–24 SNPs between the strains (Supplementary Figure S2).

**Figure 3:**
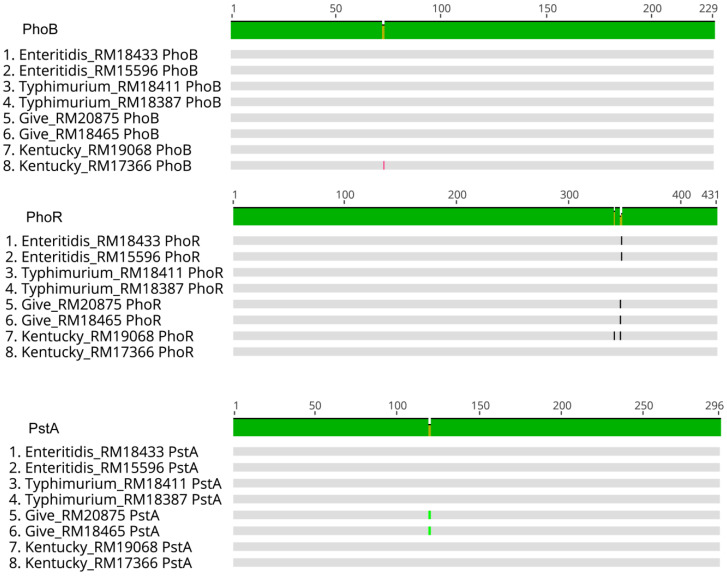
Alignments of predicted protein sequences of PhoB, PhoR, and PstA. Protein sequences were predicted from DNA sequences of the strains indicated. Strains are labeled with the serovar and their RM number. Disagreements in protein sequence are indicated by a gap in the green consensus bar and are shown by a line in the corresponding site in the individual sequences. Green line indicates consensus between the strains. Different color vertical lines in the individual strain sequences indicate amino acid identities between strains.

The remaining three genes involved in Pi metabolism (*phoB*, *phoR*, and *pstA*) had differences in the nucleic acid and in the predicted protein sequences between the eight strains and four serovars (Figure 3; Supplementary Figures S1 and S2). The *phoBR* and *pstA* genes were the same size, with *phoB* at 690 bp, *phoR* at 1296 bp, and *pstA* at 891 bp. The ranges of SNP differences were 0–11 for *phoB*, 0–13 for *phoR,* and 0–24 for *pstA*. The predicted protein sequences of the transcription regulator PhoB were identical in seven of the eight strains; the sole difference was *S.* Kentucky RM17366, which had a Met73Leu substitution. The predicted amino acid sequence of *pstA* varied only for the *S.* Give strains RM18465 and RM20875, which each had an Ile120Val substitution. This variant would result in a conserved substitution in this inner membrane protein responsible for Pi translocation across the membrane [46]. The gene with the most predicted amino acid substitutions was the histidine kinase *phoR* (Figure 3). There were four different PhoR protein sequences among the eight strains. The two *S.* Typhimurium strains and one of the *S.* Kentucky strains (RM17366) shared identical PhoR amino acid sequences. Both *S.* Enteritidis strains had an Ala347Val substitution of this sequence. The two *S*. Give strains and *S*. Kentucky strain RM19068 had a Val346Ala substitution, and RM19068 had an additional Thr340Lys substitution. The valine–alanine substitutions were conservative amino acid substitutions. Threonine–lysine resulted in R groups with a charge difference (neutral—basic, respectively). All of the amino acid changes in the predicted PhoR sequence were in the catalytic ATP-binding (CA) domain of the protein responsible for autophosphorylation of this sensor histidine kinase [47].

**Figure 4:**
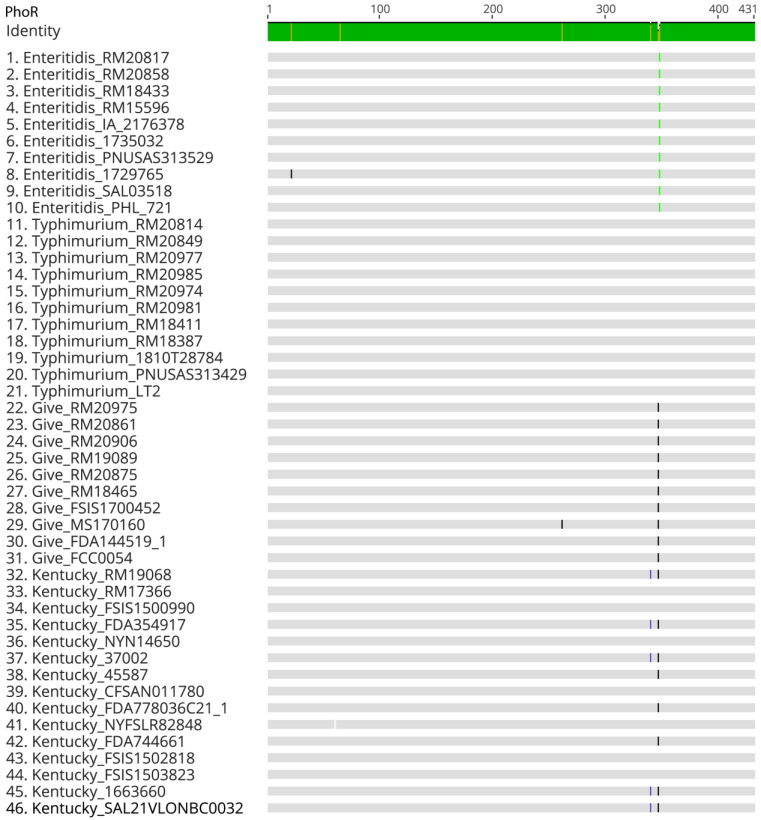
Alignment of PhoR amino acid sequences from 46 Salmonella strains. Green line indicates consensus between the strains. Different color vertical lines in the individual strain sequences indicate amino acid identities between strains.

To determine if these PhoR amino acid differences between serovars were present in additional *Salmonella* genomes, additional PhoR sequences were compared from the genomes of serovars Enteritidis, Give, Kentucky, and Typhimurium. Including the 8 strains highlighted in Figure 3, the PhoR sequences from 10 Enteritidis strains, 10 Give strains, 15 Kentucky strains, and 11 Typhimurium strains were compared (Figure 4). All the *S.* Typhimurium PhoR sequences were identical to each other. All the *S.* Enteritidis had the same Ala347Val substation indicated above (Figure 3), and 9 of the 10 were identical. *S.* Enteritidis strain 1729765 contained a Leu22Phe, which is a conserved substitution. Nine of the *S.* Give PhoR sequences were identical and contained the Val346Ala substitution indicated above (Figure 3). The final *S.* Give strain (MS170160) also contained the Val346Ala in addition to a Phe261Leu substitution. While the PhoR sequences were mostly conserved within the serovars Enteritidis, Give, and Typhimurium, there were three different PhoR sequences among the *S.* Kentucky strains. Seven *S.* Kentucky strains (RM17366, FSIS1500990, NY-NY14650, CFSAN011780, NY-FSL-R8-2848, FSIS1502818, and FSIS1503823) had PhoR sequences that were identical to the *S.* Typhimurium strains (except for a deletion of Pro64 in NY-FSL-R8-2848). Interestingly, these seven strains belong to Sequence Type 152. Three *S.* Kentucky strains (45587, FDA778036 C2 1-1, and FDA744661) had the Val346Ala substitution observed in the *S.* Give strains. These three strains belonged to ST696, ST314, and ST314, respectively. Finally, five *S.* Kentucky strains (RM19068, FDA354917, 37002, 1663660, and SAL-21-VL-ON-BC-0032) contained the Val346Ala and Thr340Lys substitutions. These four strains were all ST198.

## 4. Discussion

While nutritional differences have long been used to distinguish species of bacteria from each other, such differences are also described within species. Strains of *S.* serovar Paratyphi are distinguished by isolates that can and cannot ferment D-tartrate [48]. Enrichment culture was developed to favor growth of a target organism and limit growth of potential competitors for the same nutrient sources in a culture medium, but it will always be a competition for nutrients between the microbiota present in the inoculating sample. One reason for the adoption of culture-independent methods for microbial community analysis is because of issues of bias in various enrichment media. Direct comparisons of culture-dependent and culture-independent methods reveal large differences in community breakdowns between the two methods in vastly different ecosystems, including enumeration of antibiotic-resistant bacteria in surface waters, characterizations of plant endophytic microbiota, and dental water systems [49,50,51]. However, in public health laboratories that conduct surveillance for foodborne pathogens, the isolation of individual strains is still important. Metagenomics methods for surveillance are beginning to be discussed and developed [52,53], but there is still a need for bacterial isolates for subtyping and inclusion in national databases to link foodborne illness and outbreaks [54].

In assessing factors that might affect fitness in culture media, we studied strains of four different *Salmonella* serovars. In pure cultures, growth kinetics were similar for the serovars in TSB and BPW. While the Enteritidis and Typhimurium strains had generation times about 10% faster than Kentucky and Give, this was within standard error. However, such slight differences might affect fitness when in mixed cultures, and this is a subject of further study. Higher levels of growth in TSB reflect the fact that TSB is a richer medium than BPW. BPW contains peptone (10 g/L), NaCl, and sodium and potassium phosphates for a phosphate concentration of approximately 36 mM [14]. Peptone is a complex medium made from the hydrolysis of animal proteins. TSB comprises peptides sourced from digests of casein (15 g/L) and soybean (5 g/L), NaCl, 14 mM of glucose, and 14 mM of potassium phosphate [14]. The protein hydrolysates serve as the sources of carbon, nitrogen, sulfur, and energy [14].

We used phenotype microarrays to determine potential nutritional differences, and there were over 150 phenotype differences noted. It is interesting that Enteritidis strains were most adept at using dipeptides as nitrogen sources. *S.* Enteritidis strains are often outgrown by other *Salmonella* serovars when in mixed serovar cultures. Deaven et al. showed that strains of serovars Enteritidis and Typhimurium were masked in 71 and 78% of samples, respectively, when they were detected in fresh water samples [9]. In samples of naturally contaminated chicken carcasses, Cox et al. revealed that serovar Enteritidis could be detected after enrichments in Buffered Peptone Water from three of the eight carcasses using the molecular method CRISPR-SeroSeq but was found through culture-dependent methods only once [7]. Enteritidis and Typhimurium strains both displayed the potential to grow on various sugars such as mannitol, xylose, rhamnose, while Typhimurium strains utilized tagatose. Growth on these sugars has been documented for *Salmonella* Typhimurium previously, and xylose is used in *Salmonella* selective plating media, including Xylose Lysine Desoxycholate Agar and Xylose Lysine Tergitol 4 Agar [55,56,57,58,59]. Addition of any of these sugars to BPW or other complex pre-enrichment media might aid in improving the growth of Enteritidis and Typhimurium strains when competing with serovar Kentucky and/or Give strains in enrichments; this will be addressed in future studies.

The number of different compounds that may be used as phosphorus sources and the better growth with the individual phosphorus sources by *S.* Kentucky strains is curious. Phosphorus is usually assimilated by bacteria in the form of inorganic phosphates (Pi) [60]. Phosphorus is not predominant in nature, which is why excess phosphates (as well as nitrates and sulfates) in agricultural and sewage runoff lead to eutrophication in surface waters and grasslands [61]. The concentration of phosphates used in laboratory culture media is artificial, and many bacteria are stressed or inhibited by high phosphate concentrations [62,63,64]. *S.* Kentucky might be better at scavenging for phosphorus in various conditions. Therefore, we thought a closer look at phosphate metabolism in these strains might be useful.

Of the nine genes assessed, the gene product that showed differences based on serovar was *phoR*. The PhoB/PhoR two-component regulatory signal transduction system regulates the expression of genes involved in the transport of Pi and other P-containing compounds [65]. These are not the only genes comprising the Pho regulon, which is estimated to contain at least 137 gene products in *Escherichia coli* [65,66]. Proteomic analysis of the *S.* Typhimurium strain 14028s revealed over 300 proteins that are regulated by Pi-limiting conditions, and at least 27 of these are regulated by PhoB [26]. PhoR autophosphorylates upon reduced Pi levels and transfers that phosphoryl group to PhoB, thereby activating PhoB. When Pi levels are sufficient, PhoR removes the phosphoryl group from PhoB [29]. It was reported recently that *Salmonella* limits Pi uptake to maintain cellular processes involving Mg^2+^ homeostasis. Excess Pi uptake leads to increased ATP, which in turn chelates intracellular Mg^2+^ [28]. Therefore, regulation of aspects of cell metabolism that control nutrient assimilation, cellular processes, and survival of stresses and transition into the stationary phase are complex. Sequence variability in a central two-component regulatory system might result in slight differences in functionality of the proteins. The increased variability in *phoR* compared to the other genes led to examination of more *phoR* gene sequences from additional *Salmonella* strains.

The highest *phoR* sequence variability was among the *S.* Kentucky strains. *S.* Kentucky is a polyphyletic serovar with at least two different subgroups [67], and our comparison of 15 *S.* Kentucky strains found three different predicted protein sequences for PhoR. Interestingly, *S.* Kentucky ST152 and ST198 each had unique PhoR substitutions. In a study of another polyphyletic serovar (*S.* Mississippi), Cheng et al. concluded that the O and H antigenic elements that determine serovar may be inherited from different ancestors, leading to different sequence types with the same serovar [68]. More study of the phylogenetically distinct *S.* Kentucky strains is needed to determine if similar independent acquisitions of antigenic elements occurred.

Nearly all of the variability in PhoR sequences was in the CA domain, i.e., the part of the protein involved in ATP binding and catalysis. This region is located between amino acid residues 267 and 431. Immediately upstream of the CA domain is the DHp domain (located between amino acid residues 193 and 267), which is responsible for dimerization and histidine phosphorylation. The autophosphorylation of PhoR involves the transfer of a phosphoryl group from the CA domain to an essential His-213 residue in the DHp domain [47]. The PhoR protein has opposing kinase and phosphatase functions in the CA and DHp domains, and it was predicted that these two activities are controlled by physical constraint and a swing movement in the protein to prevent access of the CA domain to the DHp domain [65]. Such constraint between the domains has been described in studies of the crystal structure of similar sensor histidine kinases [69,70]. The mechanism by which PhoR receives the environmental phosphate signal is unknown [46], but in *Salmonella,* the *phoBR* operon is partly responsible for maintaining intracellular phosphate levels between 1 and 10 mM [65,71,72]. Phosphate homeostasis, in turn, involves multiple functions in the cell [26,28,65]. While the different PhoR proteins in these *Salmonella* are obviously functional (as the operon is essential), it might be possible that sequence differences in this CA domain have variable kinetics. We could not find any studies in the literature that involved direct testing of amino acid variations in the CA domain of PhoR, but it is tempting to speculate that changes in this region might affect the efficiency of function. Because of the complexity of the interactions of the proteins involved in phosphate metabolism and transport as well as the central role phosphate homeostasis plays in cellular metabolism, small differences in efficiency might affect competition between strains when in complex culture backgrounds.

In addition to the nutritional and genomic differences reported here, there are other factors beyond culture media that could contribute to unequal enrichment of *Salmonella* serovars. *Salmonella* strains were shown to enter into a viable but non-culturable (VBNC) state when incubated on dried fruit [73]. While a cocktail of *Salmonella* strains was not detectable via enrichment culture from dried apple slices after 46 days of incubation, live/dead staining showed 25% and 32% of the total population were viable after storage for 50 days at 25 °C and 110 days at 4 °C, respectively [73]. Bacteria can enter the VBNC state after exposure to stresses such as temperature, sanitizers, osmotic pressure, pH changes, and nutrient starvation [74,75,76]. Similar conditions may simply lead to stressed cells that are less likely to survive enrichment [77]. Several of these conditions may be encountered when *Salmonella* is present as a contaminant in and on foods. Additionally, *Salmonella* can survive inside of protozoa, which might mask its presence and render it more resistant to disinfectants [74,78]. In enrichment culture, the target organism must compete with other microbes present on or in the foods. This natural microbiota can vary depending on several factors, including the type of food, location, time of year, or natural variation [79]. Similarly, other microorganisms present in food samples may produce compounds such as bacteriocins that kill *Salmonella* [80]. Concentration effects of different *Salmonella* serovars that may contaminate the same food product may also affect the serovars that result after the enrichment process. *S.* Kentucky and *S.* Give may dominate enrichment cultures simply because there are many more of those types in the samples being tested. Surveillance for pathogens in foods is a complex process with many variables that affect the outcome. The comparison of different *Salmonella* serovars in growth characteristics is the beginning of a process to determine if the methods might be altered to achieve a fuller picture of the subtypes that might be present in contaminated foods.

## 5. Conclusions

To assess differential retrieval of *Salmonella* serovars in enrichment cultures, this study tested growth of four different serovars of *Salmonella* and revealed similar growth kinetics for the strain in pure cultures in TSB and BPW. Nutrient utilization differences between the serovars provided a basis of experimentation for supplements to enrichment culture media to enhance recovery of serovars of clinical interest that might be missed in routine food surveillance. In-depth analysis of predicted protein sequences of genes involved in phosphorus metabolism indicated differences in *phoR* sequence among the serovars. Further experimentation regarding the efficiency of various PhoR protein sequences may shed light on the efficiency of nutrient metabolism. Variations in the outcomes of enrichment culture may be affected by many factors, and small populations of specific subtypes can be missed by culture-based methods. Molecular and/or immunogenic methods that detect living cells may be better suited to finding small numbers of contaminants in situations where isolated colonies are not necessary.

## Figures and Tables

**Table 1:** *Salmonella* strains used in this study.

Strain ID	Serovar	Sequence Type	Source, Year
RM18465 *^,1^	Give	654	Water/sediment, 2014
RM20875 *	Give	158	Water/sediment, 2013
RM15596 *	Enteritidis	814	Water/sediment, 2011
RM15890	Enteritidis	ND ^2^	Water/sediment, 2011
RM16950	Enteritidis	ND	Water/sediment, 2011
RM18433 *	Enteritidis	11	Water/sediment, 2013
RM20787	Enteritidis	ND	Water/sediment, 2015
RM1316	Kentucky	ND	Ground beef, 1997
RM7890	Kentucky	ND	Ground chicken, 2008
RM17366 *	Kentucky	152	Water/sediment, 2013
RM19068 *	Kentucky	198	Water/sediment, 2015
RM17930	Typhimurium	ND	Water/sediment, 2013
RM16783	Typhimurium	ND	Water/sediment, 2012
RM18411 *	Typhimurium	19 ^3^	Water/sediment, 2014
RM18387 *	Typhimurium	19 ^3^	Water/sediment, 2014

^1^ Strains marked with an asterisk (*) were subjected to whole genome sequencing. ^2^ ND: not determined. ^3^ While these are the same sequence type, their whole genome MLST profile is different, indicating they are different strains.

**Table 2:** Genes involved in phosphate metabolism in *Salmonella*.

Gene/Operon	Identity	Function
*phoBR*	Two-component histidine kinase response regulatory proteins	Respond to environmental Pi levels and transcriptional regulation of genes involved in phosphate transfer and utilization
*pstSCAB*	Phosphate ABC transporter	High-affinity Pi transporter
*phoU*	Phosphate-specific transport system accessory	Binds to and controls function of PstSCAB transfer system Binds Mg^2+^ and Mn^2+^
*rpoS*	Sigma factor S	Cellular responses to stresses
*iraP*	RpoS adaptor protein	Inhibits RpoS proteolysis during Pi starvation
*rrsB*	Adaptor protein	Regulator of RpoS degradation

**Table 3:** Growth kinetics of *Salmonella* serovars in TSB and BPW at 37 °C.

Medium	Serovar	Lag Phase Length (h)	Generation Time (h)	Maximum CFU/mL
TSB ^1^	Enteritidis	3.0	0.51	1.32 × 10^10^
	Give	2.3	0.58	2.83 × 10^10^
	Kentucky	2.2	0.56	3.23 × 10^10^
	Typhimurium	2.2	0.49	3.11 × 10^10^
BPW ^1^	Enteritidis	2.3	0.50	6.69 × 10^9^
	Give	2.2	0.57	6.93 × 10^9^
	Kentucky	2.2	0.57	8.07 × 10^9^
	Typhimurium	2.3	0.51	7.95 × 10^9^

^1^ TSB: Trypticase Soy Broth; BPW: Buffered Peptone Water.

## Data Availability

Genome sequence data were deposited with the NCBI under the BioProject ID PRJNA875079. The raw sequencing data were sent to the Sequence Read Archive and are identified by BioSample accession numbers SAMN30597303–SAMN30597310. Strains were identified by their ID numbers: RM15596, RM17366, RM18387, RM18411, RM18433, RM18465, RM19068, and RM20875.

## Supplementary Materials

The following supporting information can be downloaded at: https://www.mdpi.com/article/10.3390/microorganisms11082109/s1, Table S1: Salmonella genomes used in this study; Table S2: Heat map of all 157 phenotype differences between the strains and serovars; Figure S1: Comparisons of nine protein sequences between sequenced strains in this study; Figure S2: Comparisons of nine gene sequences between sequenced strains in this study.
