# Supplementary material for: Comparison of Phenotype Nutritional Profiles and Phosphate Metabolism Genes in Four Serovars of Salmonella enterica from Water Sources
Source: Microorganisms. 2023 Aug 18;11(8):2109. doi: 10.3390/microorganisms11082109 (PMC10459026; doi:10.3390/microorganisms11082109)
Supplement: Supplementary file 1 [file microorganisms-11-02109-s001.zip › microorganisms-2467393-supplementary/Supplementary Figure S1 - aa diffs.pdf]

Supplementary Figure S1. Comparisons of nine protein sequences between sequenced strains in this study. Heat maps indicating amino acid differences are shown for the proteins. The darker the cell the more similarity between the indicated strains. Colorations are not comparable between heat maps.

PhoB

	Enteritidis_RM18433	Enteritidis_RM15596	Typhimurium_RM18411	Typhimurium_RM18387	Give_RM20875	Give_RM18465	Kentucky_RM19068	Kentucky_RM17366
Enteritidis_RM18433		0	0	0	0	0	0	1
Enteritidis_RM15596	0		0	0	0	0	0	1
Typhimurium_RM18411	0	0		0	0	0	0	1
Typhimurium_RM18387	0	0	0		0	0	0	1
Give_RM20875	0	0	0	0		0	0	1
Give_RM18465	0	0	0	0	0		0	1
Kentucky_RM19068	0	0	0	0	0	0		1
Kentucky_RM17366	1	1	1	1	1	1	1	

PhoR

	Enteritidis_RM18433	Enteritidis_RM15596	Typhimurium_RM18411	Typhimurium_RM18387	Give_RM20875	Give_RM18465	Kentucky_RM19068	Kentucky_RM17366
Enteritidis_RM18433		0	1	1	2	2	3	1
Enteritidis_RM15596	0		1	1	2	2	3	1
Typhimurium_RM18411	1	1		0	1	1	2	0
Typhimurium_RM18387	1	1	0		1	1	2	0
Give_RM20875	2	2	1	1		0	1	1
Give_RM18465	2	2	1	1	0		1	1
Kentucky_RM19068	3	3	2	2	1	1		2
Kentucky_RM17366	1	1	0	0	1	1	2	

PstA

	Enteritidis_RM18433	Enteritidis_RM15596	Typhimurium_RM18411	Typhimurium_RM18387	Give_RM20875	Give_RM18465	Kentucky_RM19068	Kentucky_RM17366
Enteritidis_RM18433		0	0	0	1	1	0	0
Enteritidis_RM15596	0		0	0	1	1	0	0
Typhimurium_RM18411	0	0		0	1	1	0	0
Typhimurium_RM18387	0	0	0		1	1	0	0
Give_RM20875	1	1	1	1		0	1	1
Give_RM18465	1	1	1	1	0		1	1
Kentucky_RM19068	0	0	0	0	1	1		0
Kentucky_RM17366	0	0	0	0	1	1	0	

PhoU

	Enteritidis_RM18433	Enteritidis_RM15596	Typhimurium_RM18411	Typhimurium_RM18387	Give_RM20875	Give_RM18465	Kentucky_RM19068	Kentucky_RM17366
Enteritidis_RM18433		0	0	0	0	0	0	0
Enteritidis_RM15596	0		0	0	0	0	0	0
Typhimurium_RM18411	0	0		0	0	0	0	0
Typhimurium_RM18387	0	0	0		0	0	0	0
Give_RM20875	0	0	0	0		0	0	0
Give_RM18465	0	0	0	0	0		0	0
Kentucky_RM19068	0	0	0	0	0	0		0
Kentucky_RM17366	0	0	0	0	0	0	0	

PstB

	Enteritidis_RM18433	Enteritidis_RM15596	Typhimurium_RM18411	Typhimurium_RM18387	Give_RM20875	Give_RM18465	Kentucky_RM19068	Kentucky_RM17366
Enteritidis_RM18433		0	0	0	0	0	0	0
Enteritidis_RM15596	0		0	0	0	0	0	0
Typhimurium_RM18411	0	0		0	0	0	0	0
Typhimurium_RM18387	0	0	0		0	0	0	0
Give_RM20875	0	0	0	0		0	0	0
Give_RM18465	0	0	0	0	0		0	0
Kentucky_RM19068	0	0	0	0	0	0		0
Kentucky_RM17366	0	0	0	0	0	0	0	

PstC

	Enteritidis_RM18433	Enteritidis_RM15596	Typhimurium_RM18411	Typhimurium_RM18387	Give_RM20875	Give_RM18465	Kentucky_RM19068	Kentucky_RM17366
Enteritidis_RM18433		0	0	0	0	0	0	0
Enteritidis_RM15596	0		0	0	0	0	0	0
Typhimurium_RM18411	0	0		0	0	0	0	0
Typhimurium_RM18387	0	0	0		0	0	0	0
Give_RM20875	0	0	0	0		0	0	0
Give_RM18465	0	0	0	0	0		0	0
Kentucky_RM19068	0	0	0	0	0	0		0
Kentucky_RM17366	0	0	0	0	0	0	0	

IraP

	Enteritidis_RM18433	Enteritidis_RM15596	Typhimurium_RM18411	Typhimurium_RM18387	Give_RM20875	Give_RM18465	Kentucky_RM19068	Kentucky_RM17366
Enteritidis_RM18433		0	0	0	0	0	0	0
Enteritidis_RM15596	0		0	0	0	0	0	0
Typhimurium_RM18411	0	0		0	0	0	0	0
Typhimurium_RM18387	0	0	0		0	0	0	0
Give_RM20875	0	0	0	0		0	0	0
Give_RM18465	0	0	0	0	0		0	0
Kentucky_RM19068	0	0	0	0	0	0		0
Kentucky_RM17366	0	0	0	0	0	0	0	

RpoS

	Enteritidis_RM18433	Enteritidis_RM15596	Typhimurium_RM18411	Typhimurium_RM18387	Give_RM20875	Give_RM18465	Kentucky_RM19068	Kentucky_RM17366
Enteritidis_RM18433		0	0	0	0	0	0	0
Enteritidis_RM15596	0		0	0	0	0	0	0
Typhimurium_RM18411	0	0		0	0	0	0	0
Typhimurium_RM18387	0	0	0		0	0	0	0
Give_RM20875	0	0	0	0		0	0	0
Give_RM18465	0	0	0	0	0		0	0
Kentucky_RM19068	0	0	0	0	0	0		0
Kentucky_RM17366	0	0	0	0	0	0	0	

RrsB

	Enteritidis_RM18433	Enteritidis_RM15596	Typhimurium_RM18411	Typhimurium_RM18387	Give_RM20875	Give_RM18465	Kentucky_RM19068	Kentucky_RM17366
Enteritidis_RM18433		0	0	0	0	0	0	0
Enteritidis_RM15596	0		0	0	0	0	0	0
Typhimurium_RM18411	0	0		0	0	0	0	0
Typhimurium_RM18387	0	0	0		0	0	0	0
Give_RM20875	0	0	0	0		0	0	0
Give_RM18465	0	0	0	0	0		0	0
Kentucky_RM19068	0	0	0	0	0	0		0
Kentucky_RM17366	0	0	0	0	0	0	0	
