# Supplementary material for: Comparison of Phenotype Nutritional Profiles and Phosphate Metabolism Genes in Four Serovars of Salmonella enterica from Water Sources
Source: Microorganisms. 2023 Aug 18;11(8):2109. doi: 10.3390/microorganisms11082109 (PMC10459026; doi:10.3390/microorganisms11082109)
Supplement: Supplementary file 1 [file microorganisms-11-02109-s001.zip › microorganisms-2467393-supplementary/Supplementary Figure S2 - SNP diffs.pdf]

Supplementary Figure S2. Comparisons of nine gene sequences between sequenced strains in this study. Heat maps indicating SNP differences are shown . The darker the cell the more similarity between the indicated strains. Colorations are not comparable between heat maps.

*phoB*

| | Enteritidis_RM18433 | Enteritidis_RM15596 | Typhimurium_RM18411 | Typhimurium_RM18387 | Give_RM20875 | Give_RM18465 | Kentucky_RM19068 | Kentucky_RM17366 |
|---|---|---|---|---|---|---|---|---|
| Enteritidis_RM18433 | | 0 | 0 | 0 | 4 | 4 | 8 | 3 |
| Enteritidis_RM15596 | 0 | | 0 | 0 | 4 | 4 | 8 | 3 |
| Typhimurium_RM18411 | 0 | 0 | | 0 | 4 | 4 | 8 | 3 |
| Typhimurium_RM18387 | 0 | 0 | 0 | | 4 | 4 | 8 | 3 |
| Give_RM20875 | 4 | 4 | 4 | 4 | | 0 | 8 | 7 |
| Give_RM18465 | 4 | 4 | 4 | 4 | 0 | | 8 | 7 |
| Kentucky_RM19068 | 8 | 8 | 8 | 8 | 8 | 8 | | 11 |
| Kentucky_RM17366 | 3 | 3 | 3 | 3 | 7 | 7 | 11 | |

*phoR*

| | Enteritidis_RM18433 | Enteritidis_RM15596 | Typhimurium_RM18411 | Typhimurium_RM18387 | Give_RM20875 | Give_RM18465 | Kentucky_RM19068 | Kentucky_RM17366 |
|---|---|---|---|---|---|---|---|---|
| Enteritidis_RM18433 | | 0 | 3 | 3 | 10 | 10 | 12 | 6 |
| Enteritidis_RM15596 | 0 | | 3 | 3 | 10 | 10 | 12 | 6 |
| Typhimurium_RM18411 | 3 | 3 | | 0 | 11 | 11 | 13 | 7 |
| Typhimurium_RM18387 | 3 | 3 | 0 | | 11 | 11 | 13 | 7 |
| Give_RM20875 | 10 | 10 | 11 | 11 | | 0 | 10 | 8 |
| Give_RM18465 | 10 | 10 | 11 | 11 | 0 | | 10 | 8 |
| Kentucky_RM19068 | 12 | 12 | 13 | 13 | 10 | 10 | | 10 |
| Kentucky_RM17366 | 6 | 6 | 7 | 7 | 8 | 8 | 10 | |

*pstA*

| | Enteritidis_RM18433 | Enteritidis_RM15596 | Typhimurium_RM18411 | Typhimurium_RM18387 | Give_RM20875 | Give_RM18465 | Kentucky_RM19068 | Kentucky_RM17366 |
|---|---|---|---|---|---|---|---|---|
| Enteritidis_RM18433 | | 1 | 7 | 7 | 19 | 19 | 15 | 14 |
| Enteritidis_RM15596 | 1 | | 8 | 8 | 20 | 20 | 16 | 15 |
| Typhimurium_RM18411 | 7 | 8 | | 0 | 24 | 24 | 8 | 17 |
| Typhimurium_RM18387 | 7 | 8 | 0 | | 24 | 24 | 8 | 17 |
| Give_RM20875 | 19 | 20 | 24 | 24 | | 0 | 24 | 19 |
| Give_RM18465 | 19 | 20 | 24 | 24 | 0 | | 24 | 19 |
| Kentucky_RM19068 | 15 | 16 | 8 | 8 | 24 | 24 | | 19 |
| Kentucky_RM17366 | 14 | 15 | 17 | 17 | 19 | 19 | 19 | |

*phoU*

| | Enteritidis_RM18433 | Enteritidis_RM15596 | Typhimurium_RM18411 | Typhimurium_RM18387 | Give_RM20875 | Give_RM18465 | Kentucky_RM19068 | Kentucky_RM17366 |
|---|---|---|---|---|---|---|---|---|
| Enteritidis_RM18433 | | 0 | 18 | 18 | 24 | 24 | 17 | 19 |
| Enteritidis_RM15596 | 0 | | 18 | 18 | 24 | 24 | 17 | 19 |
| Typhimurium_RM18411 | 18 | 18 | | 0 | 20 | 20 | 9 | 9 |
| Typhimurium_RM18387 | 18 | 18 | 0 | | 20 | 20 | 9 | 9 |
| Give_RM20875 | 24 | 24 | 20 | 20 | | 0 | 21 | 21 |
| Give_RM18465 | 24 | 24 | 20 | 20 | 0 | | 21 | 21 |
| Kentucky_RM19068 | 17 | 17 | 9 | 9 | 21 | 21 | | 10 |
| Kentucky_RM17366 | 19 | 19 | 9 | 9 | 21 | 21 | 10 | |

*pstB*

| | Enteritidis_RM18433 | Enteritidis_RM15596 | Typhimurium_RM18411 | Typhimurium_RM18387 | Give_RM20875 | Give_RM18465 | Kentucky_RM19068 | Kentucky_RM17366 |
|---|---|---|---|---|---|---|---|---|
| Enteritidis_RM18433 | | 0 | 3 | 3 | 6 | 6 | 7 | 5 |
| Enteritidis_RM15596 | 0 | | 3 | 3 | 6 | 6 | 7 | 5 |
| Typhimurium_RM18411 | 3 | 3 | | 0 | 5 | 5 | 6 | 4 |
| Typhimurium_RM18387 | 3 | 3 | 0 | | 5 | 5 | 6 | 4 |
| Give_RM20875 | 6 | 6 | 5 | 5 | | 0 | 9 | 7 |
| Give_RM18465 | 6 | 6 | 5 | 5 | 0 | | 9 | 7 |
| Kentucky_RM19068 | 7 | 7 | 6 | 6 | 9 | 9 | | 8 |
| Kentucky_RM17366 | 5 | 5 | 4 | 4 | 7 | 7 | 8 | |

*pstC*

| | Enteritidis_RM18433 | Enteritidis_RM15596 | Typhimurium_RM18411 | Typhimurium_RM18387 | Give_RM20875 | Give_RM18465 | Kentucky_RM19068 | Kentucky_RM17366 |
|---|---|---|---|---|---|---|---|---|
| Enteritidis_RM18433 | | 0 | 0 | 0 | 16 | 16 | 14 | 7 |
| Enteritidis_RM15596 | 0 | | 0 | 0 | 16 | 16 | 14 | 7 |
| Typhimurium_RM18411 | 0 | 0 | | 0 | 16 | 16 | 14 | 7 |
| Typhimurium_RM18387 | 0 | 0 | 0 | | 16 | 16 | 14 | 7 |
| Give_RM20875 | 16 | 16 | 16 | 16 | | 0 | 12 | 11 |
| Give_RM18465 | 16 | 16 | 16 | 16 | 0 | | 12 | 11 |
| Kentucky_RM19068 | 14 | 14 | 14 | 14 | 12 | 12 | | 9 |
| Kentucky_RM17366 | 7 | 7 | 7 | 7 | 11 | 11 | 9 | |

*iraP*

| | Enteritidis_RM18433 | Enteritidis_RM15596 | Typhimurium_RM18411 | Typhimurium_RM18387 | Give_RM20875 | Give_RM18465 | Kentucky_RM19068 | Kentucky_RM17366 |
|---|---|---|---|---|---|---|---|---|
| Enteritidis_RM18433 | | 0 | 0 | 0 | 0 | 0 | 0 | 0 |
| Enteritidis_RM15596 | 0 | | 0 | 0 | 0 | 0 | 0 | 0 |
| Typhimurium_RM18411 | 0 | 0 | | 0 | 0 | 0 | 0 | 0 |
| Typhimurium_RM18387 | 0 | 0 | 0 | | 0 | 0 | 0 | 0 |
| Give_RM20875 | 0 | 0 | 0 | 0 | | 0 | 0 | 0 |
| Give_RM18465 | 0 | 0 | 0 | 0 | 0 | | 0 | 0 |
| Kentucky_RM19068 | 0 | 0 | 0 | 0 | 0 | 0 | | 0 |
| Kentucky_RM17366 | 0 | 0 | 0 | 0 | 0 | 0 | 0 | |

*rpoS*

| | Enteritidis_RM18433 | Enteritidis_RM15596 | Typhimurium_RM18411 | Typhimurium_RM18387 | Give_RM20875 | Give_RM18465 | Kentucky_RM19068 | Kentucky_RM17366 |
|---|---|---|---|---|---|---|---|---|
| Enteritidis_RM18433 | | 0 | 3 | 3 | 3 | 3 | 2 | 2 |
| Enteritidis_RM15596 | 0 | | 3 | 3 | 3 | 3 | 2 | 2 |
| Typhimurium_RM18411 | 3 | 3 | | 0 | 2 | 2 | 1 | 1 |
| Typhimurium_RM18387 | 3 | 3 | 0 | | 2 | 2 | 1 | 1 |
| Give_RM20875 | 3 | 3 | 2 | 2 | | 0 | 1 | 1 |
| Give_RM18465 | 3 | 3 | 2 | 2 | 0 | | 1 | 1 |
| Kentucky_RM19068 | 2 | 2 | 1 | 1 | 1 | 1 | | 0 |
| Kentucky_RM17366 | 2 | 2 | 1 | 1 | 1 | 1 | 0 | |

*rrsB*

| | Enteritidis_RM18433 | Enteritidis_RM15596 | Typhimurium_RM18411 | Typhimurium_RM18387 | Give_RM20875 | Give_RM18465 | Kentucky_RM19068 | Kentucky_RM17366 |
|---|---|---|---|---|---|---|---|---|
| Enteritidis_RM18433 | | 1 | 4 | 4 | 4 | 4 | 6 | 8 |
| Enteritidis_RM15596 | 1 | | 5 | 5 | 5 | 5 | 7 | 9 |
| Typhimurium_RM18411 | 4 | 5 | | 0 | 4 | 4 | 6 | 8 |
| Typhimurium_RM18387 | 4 | 5 | 0 | | 4 | 4 | 6 | 8 |
| Give_RM20875 | 4 | 5 | 4 | 4 | | 0 | 6 | 6 |
| Give_RM18465 | 4 | 5 | 4 | 4 | 0 | | 6 | 6 |
| Kentucky_RM19068 | 6 | 7 | 6 | 6 | 6 | 6 | | 2 |
| Kentucky_RM17366 | 8 | 9 | 8 | 8 | 6 | 6 | 2 | |
